# Mental health service responses to human trafficking: a qualitative study of professionals’ experiences of providing care

**DOI:** 10.1186/s12888-015-0679-3

**Published:** 2015-11-17

**Authors:** Jill Domoney, Louise M. Howard, Melanie Abas, Matthew Broadbent, Sian Oram

**Affiliations:** Institute of Psychiatry, Psychology and Neuroscience, King’s College London, De Crespigny Park, London, SE5 8AF United Kingdom; South London and Maudsley NHS Foundation Trust, Denmark Hill, London, SE5 8AZ United Kingdom

**Keywords:** Human trafficking, Mental health, Mental health services, Mental disorder, Health services research, Qualitative

## Abstract

**Background:**

Human trafficking is a global crime and human rights violation. Although research has demonstrated a high prevalence of mental disorder among trafficked people and that trafficked people are in contact with mental health services, little is known about mental health professionals’ experiences of identifying and providing care for trafficked people. This study aimed to understand how people are identified as trafficked within mental health services and the challenges professionals experience in responding to trafficked people’s mental health needs.

**Method:**

Qualitative study of electronic health records of trafficked people in contact with secondary mental health services in South East London, England. Comprehensive clinical electronic health records for over 200,000 patients in contact with secondary mental health services in South London were searched and retrieved to identify trafficked patients. Content analysis was used to establish how people were identified as trafficked, and thematic analysis was used to explore the challenges experienced in responding to mental health needs.

**Results:**

The sample included 130 trafficked patients, 95 adults and 35 children. In 43 % (41/95) of adult cases and 63 % (22/35) child cases, mental health professionals were informed that their patient was a potential victim of trafficking by another service involved in their patient’s care. Cases were also identified through patients disclosing their experiences of exploitation and abuse. Key challenges faced by staff included social and legal instability, difficulties ascertaining history, patients’ lack of engagement, availability of services, and inter-agency working.

**Conclusions:**

Training to increase awareness, encourage helpful responses, and inform staff about the available support options would help to ensure the mental health needs of trafficked people are met. Further research is needed to establish if these challenges are similar in other health settings.

**Electronic supplementary material:**

The online version of this article (doi:10.1186/s12888-015-0679-3) contains supplementary material, which is available to authorized users.

## Background

Human trafficking is defined as the recruitment and movement of people, most often through the use of deception, threat, coercion, or the abuse of vulnerability, for the purposes of exploitation. Each year hundreds of thousands of women, men, and children are moved across and within international borders to be exploited through forced sex work, domestic servitude, forced labour in industries as diverse as agriculture, construction, and fishing, and through forced criminality. Research has shown a high prevalence of depression, anxiety and post-traumatic stress disorder among survivors of human trafficking [1–4], and has highlighted the importance of providing access to mental health assessments and appropriate psychological support. Recent research has also demonstrated that mental health services are caring for survivors of human trafficking [4]. There are a number of scenarios in which healthcare professionals may come into contact with victims of trafficking. A person may present to services while they are still in the situation of exploitation, or after having escaped. The healthcare professional may detect signs that suggest exploitation or abuse, be informed by another professional that their patient is a victim of trafficking, or the patient may disclose their experiences directly to the healthcare professional [5]. However, very little is known about mental health professionals’ experiences of identifying and providing care for trafficked people. Survey and qualitative research suggests that mental health professionals lack confidence in responding appropriately to trafficked people, including how to ask about experiences of trafficking and how to make referrals to support services [6], as well as feeling under-supported by their organisations [7]. The objectives of this study were therefore to understand how people are identified as potential victims of trafficking within mental health services and the challenges that mental health professionals experience in responding to trafficked people’s needs.

## Methods

### Setting and sample

Data for this study were provided by the South London and Maudsley NHS Foundation Trust (SLaM) Biomedical Research Centre (BRC) Case Register Interactive Search (CRIS) database. The SLaM Patient Journey System (PJS), an integrated electronic clinical record used across all SLaM services that provides a comprehensive record of all clinical information recorded during patients’ contacts with SLaM [8], has over 200,000 cases which are returnable through the CRIS system. CRIS allows for searching and retrieval of anonymised full patient records, using a bespoke de-identification algorithm to ensure patient identifiers are masked [9].

Free text search terms were used to search the CRIS database for adults and children who had accessed care within SLaM between 2006 and 2012 and whose records documented concerns that they were a potential victim of trafficking. This included terms such as ‘trafficked’, ‘sex slavery’, and ‘forced labour’ (see Additional file 1 for a full list of search terms). One researcher assessed returned records for eligibility. Patients were categorised as having been trafficked if their free text clinical notes indicated that their care team believed that the patient had or may have been trafficked, for example because they were informed by the patient or a third party of experiences compatible with the definition of human trafficking, or that the patient was involved in criminal proceedings against their trafficker, was claiming asylum in relation to their experiences while trafficked, or was receiving social services or voluntary sector support as a victim of trafficking. A second researcher independently assessed the eligibility of the first 10 records and assessed an additional random sample of 10 %. Discrepancies were discussed and resolved by consensus.

### Data extraction

Free text notes, which record details of patient contacts and correspondence with other professionals involved in the patient’s care, were downloaded for each case. As some individuals’ case notes comprised many entries over several years, search terms to were used to identify and retrieve notes which referred to key themes identified a priori and during preliminary analyses. For example, to identify and retrieve information on the topic of immigration, terms such as “Home Office”, “asylum”, “appeal”, and “National Asylum Support Service” or “NASS” were used. JD read all downloaded notes and manually extracted sections of text that were relevant to the two aims of the study, which were then transferred into an Excel spreadsheet for analysis.

### Analysis

Thematic analysis was used to analyse clinicians’ notes and correspondence. This involved three stages [10]. In the first stage, a random selection of case notes were read and potential codes were noted. In the second stage the full sample of case notes were read, with relevant text extracted as described above, and an initial coding framework was developed. The framework was developed by JD, with supervision from SO. In the third stage JD collated into potential themes. Themes were iteratively checked and refined against the coded extracts and the overall data set, and a thematic map was developed to represent the data in a visual format. SO reviewed the coding and checked the thematic maps against the data.

Pilot work showed that data on how patients were identified as potential victims of trafficking were not rich enough for thematic analysis as around half of cases had previously been identified by other services and case notes therefore simply stated this as a factor in assessments. A directed content analysis approach was therefore used to address this objective [11], categorising patients according to how they were identified as potential victims of trafficking e.g. prior to contact with mental health services or during contact.

Case records for adults and children were analysed separately to reflect the division of mental health services into adult and child & adolescent services. Children were defined as individuals who were younger than 18 at the time of first contact with SLaM services.

### Ethics

Ethics approval for the research use of CRIS-derived anonymised databases was granted by an independent Research Ethics Committee (Oxfordshire C, reference 08/H0606/71) and for this study by the CRIS Oversight Committee (11/025). All quotes were reviewed by the CRIS Oversight Committee to ensure no identifiable information was included.

## Results

### Characteristics of the sample

Searches of the CRIS database returned details of 691 patients whose records included one or more trafficking search terms; 558 records were excluded during screening (for example, because they related to traffic accidents, drug trafficking, or to patients’ interest in the issue of human trafficking). The final sample included 130 patients whose records indicated that they had experienced trafficking, including 95 adults and 35 children (under 18 years).

Table 1 shows the demographic characteristics and trafficking experiences of the sample. The majority of adults were female (81.3 %) and the mean age at contact with SLaM services was 26.7 (SD 6.8, range 18–49). 58.3 % had been trafficked for sexual exploitation. Two thirds (67.6 %) of children were female and the mean age at first contact with SLaM services was 14.9 (SD 2.5, range 8–17). One third of children had been trafficked for sexual exploitation and one third for domestic servitude or other forms of exploitation. No details were available regarding type of exploitation for one fifth of the trafficked adult sample and one third of the children. Further details of the sample have been described elsewhere [4].

**Table 1:** Sample characteristics

	Total (*n =* 130)	Adults (*n =* 95)	Children (*n =* 35)
Gender			
- Female	101 (77.7)	76 (80)	25 (71.4)
- Male	29 (22.3)	19 (20)	10 (28.6)
Region of origin			
- Europe	22 (16.9)	22 (23.2)	-
- Africa	65 (50)	47 (49.5)	18 (51.4)
- Asia	27 (20.8)	16 (16.8)	11 (31.4)
- Other	13 (10)	8 (8.4)	5 (14.3)
- Unknown	3 (2.3)	2 (2.1)	1 (2.9)
Age			
- 8–11	6 (4.6)	-	6 (17.1)
- 12–15	11 (8.5)	-	11 (31.4)
- 16–17	18 (13.8)	-	18 (51.4)
- 18–25	48 (36.9)	48 (50.5)	-
- 26–33	35 (26.9)	35 (36.8)	-
- 34+	12 (9.2)	12 (12.6)	-
Type of exploitation			
- Sexual	67 (51.6)	55 (57.9)	12 (34.3)
- Domestic servitude	22 (16.9)	10 (10.5)	12 (34.3)
- Other	8 (6.2)	8 (8.4)	-
- Unknown	33 (25.4)	22 (23.2)	11 (31.4)
Route of referral			
- A&E	34 (26.2)	28 (29.5)	6 (17.1)
- GP	36 (27.7)	31 (32.6)	5 (14.3)
- Other health	29 (22.3)	22 (23.2)	7 (20)
- Police/courts	7 (5.4)	5 (5.3)	2 (5.7)
- Other	23 (17.7)	6 (6.3)	15 (42.8)
- Unknown	3 (2.3)	3 (3.2)	0

### How are people identified as trafficked within a large inner city mental health service?

#### Adults

Records indicated that in 43 % of cases (41/95) mental health professionals were informed that their patient was a potential victim of trafficking by another service involved in their patient’s care, including by a local voluntary sector provider of specialist post-trafficking support, general practitioners, police, social services, or health services in other boroughs. In 47 (49 %) cases mental health professionals became aware that their patients were potential victims of trafficking during their contact with SLaM. This included disclosure by the patient of historical experiences where there was no on-going risk and, in nine cases (9.5 %), examples where the patient disclosed recently being in an exploitative situation or where professionals suspected a case of trafficking. Examples of the type of information that led to suspicion included:

“She did allude to being made to meet people via dating sites and being forced to do things against her will. (Possibly sexual in nature)… She believes another older man may be behind the activities/coercion enforced by her boyfriend……[she] may be a victim of trafficking or forced prostitution.”

“She was living in as a domestic [worker]…but says the pay has stopped…Complains she was kept “like a slave”…I will alert police to her story…will clarify whether she was trafficked into UK.”

It was not possible to establish how a person had been identified in seven (7.3 %) cases.

#### Children

Out of 35 records, 23 (63 %) indicated that mental health professionals were made aware that the individual may be a victim of trafficking by another service, most frequently by social services, but also by general practitioners and A&E departments. Eleven (31 %) were identified as potential victims of trafficking by the mental health professional during contact. This included disclosure during assessments, as well as three cases where professionals suspected a case of trafficking from information given.

“[He] told her which stop to get off at, and gave her a photo of her Uncle, whom she’d never met and told her when she saw him she should run up to him and hug him and say “Uncle” …(this sounds very concerning and I will discuss with social worker re possibility of trafficking).”

It was not possible to establish how a person was identified as trafficked in one (2.8 %) case.

### What challenges do mental health providers experience in responding to trafficked people’s mental health needs?

#### Adults

**Fig. 1:**
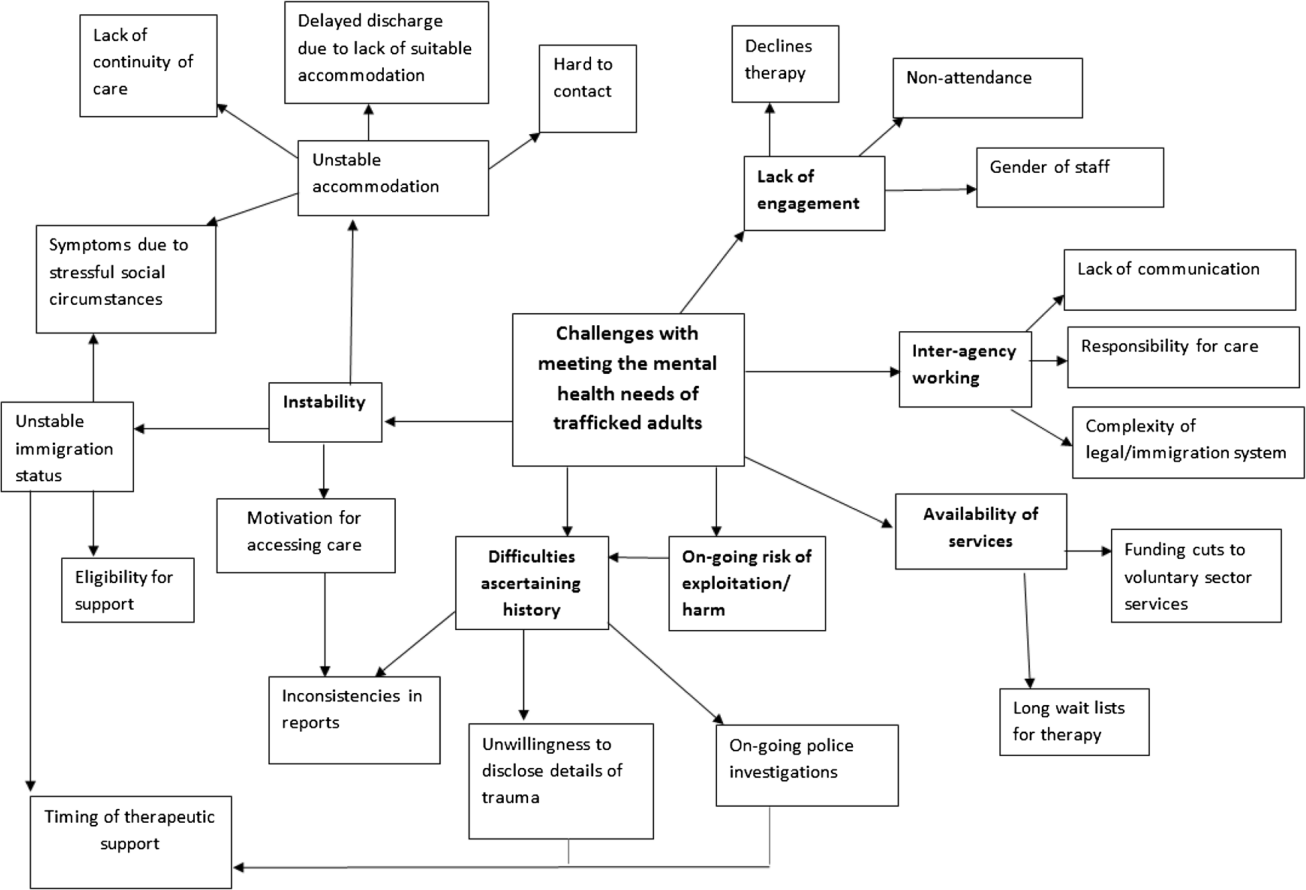
Challenges to meeting the mental health needs of trafficked adults

Figure 1 illustrates the main themes that emerged from analysis of the case notes of trafficked adults. Key themes included: social and legal instability, difficulties ascertaining history, lack of engagement, availability of services, and inter-agency working.

Records documented that many trafficked patients were living in situations of social, legal, and economic instability—particularly with regards to accommodation and immigration status—and that this posed a range of problems for mental health professionals. For example, patients being moved to accommodation outside of the service catchment area presented challenges to providing continuity of care and risked disrupting relationships between patients and professionals.

“[She] … expresses a wish not to be transferred to another [Community Mental Health Team] if possible, despite having moved out of [this] locality. This is because she does not want to have to talk over these issues with another set of professionals and have to spend time building up trust again, especially in view of the sensitive nature of her experiences.”

Continuity of care could also be interrupted where patients were moved within the service catchment area, as frequent changes of address could mean that it was difficult to stay in contact with patients.

“With no address, no clear idea on his location, no contact number there is no way of contacting [him] who was discharged yesterday.”

Uncertainties regarding the availability and entitlement to accommodation for trafficked patients could impact on the length of admission to inpatient services if placements were not available.

“[She] is agreeing to informal admission for a period of observation and investigation of her mental health needs. Home Treatment [Team] input may be difficult as she currently has nowhere to live.”

In some cases professionals queried whether patients’ motivations for accessing care were related to their need for accommodation, and raised the possibility of malingering.

“Presents with suicidal intent and auditory hallucinations. Possible motivation is accommodation”

Mental health professionals recorded concerns that this social and legal instability had a negative impact on their patient’s mental health, noting that for several patients psychological symptoms were a consequence of stressful social circumstances.

“[I] advised we would support her as much as we could but realistically we are unable to take the stress i.e. immigration issues away….Her main preoccupation is that of her immigration issues not yet being resolved which clearly impact negatively on her mental well-being.”

“Patient presented in a crisis mainly triggered by social stressors a few weeks ago. She has no recourse to public funds and lives in a small accommodation.”

In the context of ongoing social and legal stressors some professionals were reluctant to initiate psychological therapy.

“It will be difficult for [her] to engage in any long term psychotherapeutic support before she has the result of her asylum application.”

However, where there were queries over whether a patient met the diagnostic criteria for a mental health disorder or not (for example, where symptoms fluctuated in response to social stressors), this sometimes had implications for access to funding (e.g. for certain types of welfare benefits) due to enduring mental illness being classed as a disability

“Issue about funding, partly dependant on whether or not she is seen as having an enduring mental illness.”

Some professionals documented spending considerable time helping patients with social, legal and financial issues. This included writing letters to solicitors, in some cases writing reports to the Home Office in relation to an asylum claim, and assisting with organising benefits or access to other support services. However, patients’ unstable immigration status often impacted on their eligibility for other support services, hindering professionals’ efforts to improve patients’ situations.

“[She] spoke of wanting extra support from various organisations… but she was advised that she could not be helped due to her asylum status.”

“As she has ‘no recourse to public funds’, she is not able to claim welfare benefits and social housing.”

Recent or on-going traumatic experiences in relation to trafficking also presented specific challenges, including with ascertaining assessment information. This was sometimes due to ongoing police investigations which prevented clinicians from asking about traumatic events.

“I deliberately did not go into too much detail regarding her ‘journey’ to the UK as I was aware the police are investigating this.”

It was sometimes due to unwillingness by victims to discuss their experiences.

“[She] became closed off at points when I broached talking about the past, for example when she came to the UK, and said ‘I don’t want to talk about that now.’ ‘Don’t ask me any questions.’”

There were also inconsistencies in reports, leading to uncertainty about the individual’s circumstances or identity.

“New information is that [she] is known to the Home Office for coming into the country illegally. Contradicts her story of coming into the country at the age of 10.”

“There are also inconsistencies in her reports to us and some of the notes, in that an aunt is mentioned in her notes but she denied having any family here.”

A further challenge concerned lack of patient engagement with services. This included common barriers in mental health services, such as patients declining therapy or non-attendance at appointments, but also included cultural differences in understandings of mental health and issues relating to the gender of staff.

“[Social worker] mentioned that she was nervous about mental health services as culturally this was not something she was used to.”

“Did not want a male staff to assess her…she preferred any female staff. - She was informed there was no female staff at the moment.”

Service-related issues were also documented as presenting challenges to meeting the needs of potential victims of trafficking. For example, long waiting lists for therapy and cuts to funding of voluntary sector services made it difficult to access support.

“[She] was keen to receive help, and as such we have placed her on our psychology waiting list [for cognitive behavioural therapy]. Unfortunately we have a waiting list for treatment.”

“She is an asylum seeker with no recourse to public funds and her legal advice centre has been closed.”

Patients’ notes documented difficulties with inter-professional working in terms of communication and in deciding which service was responsible for care when patients were moved between boroughs.

“The split between her mental health support needs, ‘immigration status’ and the resource implications involved seem to present a hindrance to effective problem resolution and inter-professional working.”

“[She] would appear to have significant mental health concerns … However it would appear that the only link to [this borough] is an address she stayed at for a little over 2 weeks and she has more substantive connections to services in the [another borough] area.”

#### Children

**Fig. 2:**
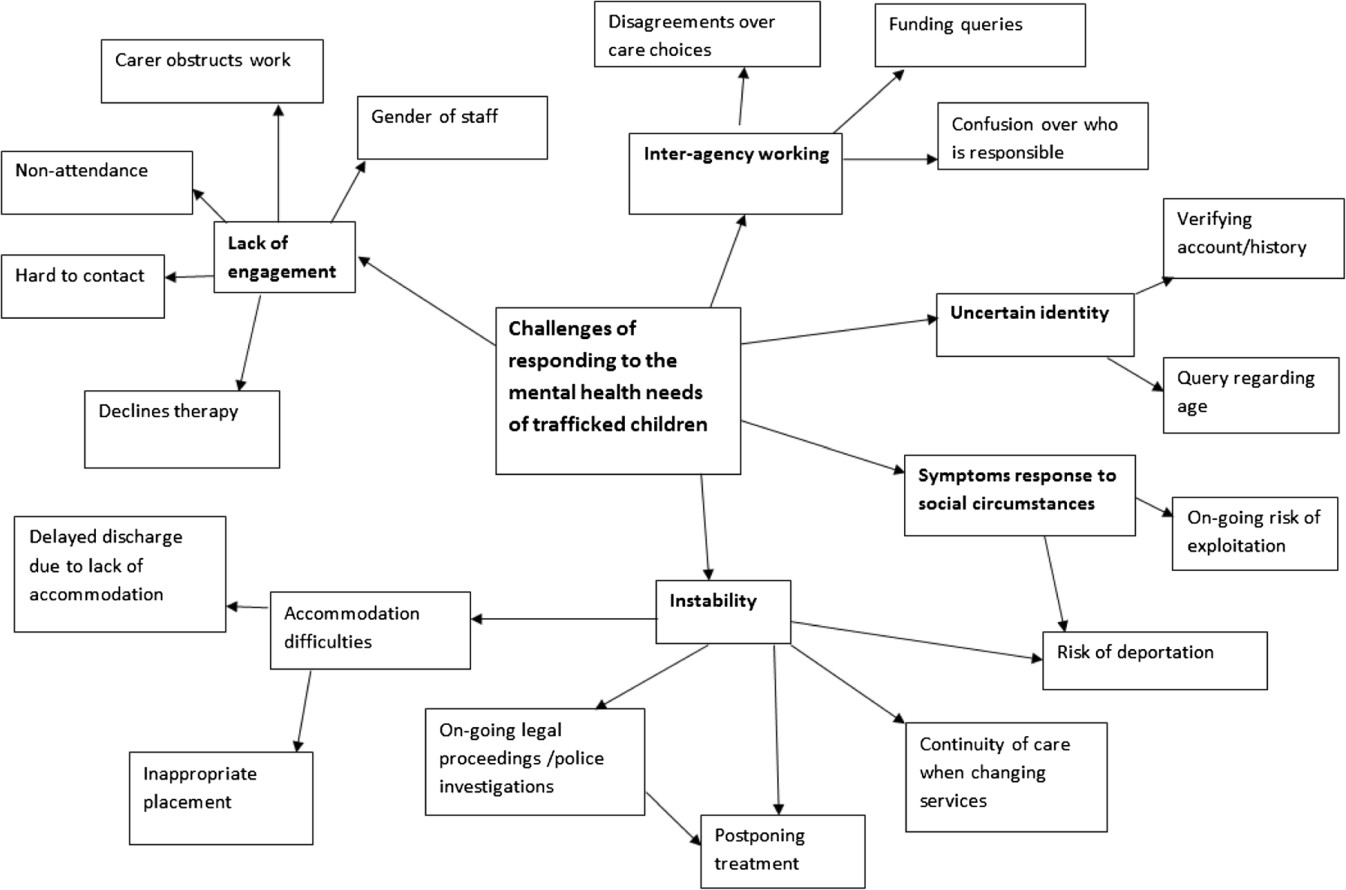
Challenges to meeting the mental health needs of trafficked children

The main themes from the analysis of the clinical notes of potential child victims of trafficking are illustrated in Fig. 2. Many of these themes overlapped with those identified in the adult data, for example, challenges due to social and legal instability, lack of engagement, inter-agency working and verifying identity. However, there were some differences. For children, the theme of instability was less to do with housing and more around immigration issues. Immigration issues didn’t present difficulties with accessing services in the same way as it did for adults, but did impact on starting therapy and led to documented increases in symptoms of stress because of the risk of deportation and on-going legal proceedings.

“According to his social worker the Home Office are actively seeking to return him to [country of origin]… Therapeutic input should be put on hold until [his] future is more secure.”

“He will be informed in the next 6–8 weeks the outcome of his right to remain in the UK. Understandably he has found the whole experience very stressful and he is not sleeping and is woken on a daily basis by nightmares and panic attacks as he is terrified he will be sent back.”

Uncertainty regarding patients’ identity and difficulties with inter-agency working were also more commonly documented in the data on potential child victims of trafficking. Issues with identity often concerned patients’ age due to decisions on whether the individual needed adult or child and adolescent services.

“She recently completed an age assessment which suggested that she was 19–21 years old…but we are awaiting a decision.”

In other cases there were references to vague, unclear patient histories, often related to parentage. This sometimes had implications for information sharing and whole family working where it was not clear what relationship adults had to the trafficked child.

“[He] was brought up by the couple but does not appear to be their biological son. He is aware that Mr F. is not his father but it is not clear whether he realises that Mrs F. is not his mother.”

Inter-professional working also presented challenges. There were examples of disagreements over care, particularly related to safety concerns, and also in relation to which service is responsible.

“I appreciate that concerns previously expressed by other professionals have been investigated. I do not understand what you then mean in saying you have never had any concerns regarding this placement.”

“CAMHS are unwilling to come to the hospital to see her and are saying that it is our responsibility and that we need to go to visit her. Explained this in not the protocol.”

Lack of engagement had similar subthemes to the adult data, including non-attendance, difficulties contacting patients, and gender of staff. However, there were also some examples of carers obstructing work, for example, through not bringing children to appointments.

“[Her foster carer] has not appeared keen to meet as [she] is thriving in the placement and she is busy so she has not seen the need.”

“[She] was referred due to reports that there was no food in the home and that she was acting as a young carer to her mother and siblings…but her parents were obstructive to CAMHS intervention and this eventually broke down after only a few months.”

## Discussion

To our knowledge, this is the first study to explore how potential victims of trafficking are identified within mental health services and the challenges that mental health professionals experience in responding to these patients’ needs.

There were few examples (nine adults and three children) of cases where previously undisclosed, recent experiences of trafficking were identified for the first time during contact with mental health services. It was therefore hard to draw conclusions about the ways in which mental health professionals identify trafficked people. Further research is needed to explore how potential victims of trafficking are identified in healthcare settings. For those where the trafficking status was already known, this information largely came from the voluntary sector, police and social services, as well as other health services and general practitioners.

Patients’ notes documented a range of challenges experienced by mental health professionals seeking to meet the needs of potential victims of trafficking. The impact of social and legal instability was a major theme across both the adult and child data. Social stressors, such as the risk of deportation and unstable housing, were seen to be a major factor causing or exacerbating symptoms, and therapeutic interventions were postponed because of uncertainty around the person’s future. The findings highlight the importance of addressing social needs when supporting recovery from mental health disorder [12]. Research has suggested that an increased number of social needs and lower levels of social support are risk factors for mental disorder among female survivors of trafficking [1]. The broader literature on depression and PTSD also indicates that ongoing social stressors can exacerbate and perpetuate symptoms [13, 14]. Records documented patients’ need for social, financial, and legal support and the efforts taken to improve the stability of patients’ circumstances as a way to support their mental health needs. This perhaps reflects a two-phased approach, whereby dealing with current threat and improving social support is prioritised before beginning therapy specific to the trauma. This is in line with guidance on the treatment of people who have experienced trauma [15, 16]. However, the data indicated that it can be challenging to take a phased, longer-term approach to care when patients who are being rehoused by the National Asylum Support Service often have interruptions in care and changes in the services providing care.

Difficulties ascertaining patient histories also spanned both child and adult data. Professionals noted inconsistencies in accounts, barriers to obtaining information, and queried accuracy, especially where this had the potential to impact on service provision. The data provided a mixed picture of whether professionals viewed inconsistencies as possible malingering for secondary gain (although these suspicions were only raised in the adult data) or as a consequence of traumatic, confusing histories. While ascertaining accurate histories may be difficult for a number of patient groups, there may be a variety of reasons why trafficked people specifically choose not to disclose information to professionals or have difficulty recalling details of their experiences. For example, they may be fearful about the risk of disclosure on their own safety or the safety of their families, or the likelihood of deportation, or be struggling with feelings of shame or guilt [17, 18]. Additionally, traumatic experiences affect memory and may impact on the individual’s ability to recall the timing, details or chronology of events [19]. Training to increase mental health professionals’ awareness of potential indicators of trafficking, the reasons for lack of disclosure, and methods to undertake sensitive assessments around trafficking experiences may support professionals in obtaining necessary information and making decision about people’s needs [12, 20]. Training programmes have been shown to increase awareness of trafficking and confidence to respond, although they have not been evaluated with respect to improved identification, referrals or care [21, 22].

Challenges relating to engagement similarly were present in both child and adult data. Some of these challenges, for example, non-attendance, are present for many patient groups [23, 24]. However, the gender of staff available for assessments seemed to be a particular issue for survivors of trafficking where experiences of sexual violence were common. Considering gender when planning assessments and booking interpreters with survivors of trafficking is important, and is more generally held to be good practice when supporting victims of violence [25].

The final themes related to service availability and inter-agency working. For adults, long waiting times for psychological therapy, funding cuts to voluntary sector services, and decisions around which service should take responsibility, presented challenges for mental health professionals. This was exacerbated by the social and legal instability experienced by trafficked patients, including dealing with immigration issues and being rehoused in a different borough. For children, issues were more generally concerned with disagreements between professionals regarding safety, as well as some difficulties deciding which service was responsible for care where children had moved between boroughs or where their age had been disputed. Age assessments can be experienced as very distressing by patients due to the implications for their sense of self and access to resources, and so this process itself has implications for mental health [26].

### Strengths and limitations

This study used an innovative methodology and data resource to access anonymised information contained in comprehensive mental health records for an otherwise hard-to-reach group. To our knowledge is the first report of qualitative analysis of free-text clinical records identified using the CRIS system. There are some limitations with the database as a source of data for qualitative research, including that professionals varied in the type and detail of information recorded. However, the study demonstrates the potential of electronic health records as a resource for qualitative research. This should be explored further in future methodological research.

A number of other limitations should be noted. Due to the search strategy used, it is possible that some individuals who would meet the definition of trafficking were missing from the sample because professionals did not enquire about or document their histories accurately. The records of some individuals comprised many entries over several years and reading every note was not feasible. In these cases search terms were developed to retrieve information about a priori themes. Although this approach may have resulted in some information being missed, only a small number of cases were not able to be categorised in terms of how the individual was identified as trafficked, and data saturation was achieved in terms of the thematic analysis of challenges experienced by mental health professionals in responding to the needs of trafficked people. There are also potential limitations in terms of the generalisability of the findings. This was an inner city setting, exploring challenges within a secondary care mental health service. Identification of and responses to trafficked people may be different in other settings and further research is needed to explore how people are identified and supported elsewhere.

## Conclusions

Mental health professionals face a number of challenges when providing care to survivors of trafficking, including dealing with social and legal instability, lack of engagement, and difficulties with inter-agency working. These challenges suggest the need for an approach which takes into account both social and psychological factors when responding to mental health needs, as well as improved communication between services. Training for professionals to increase awareness of trafficking, to support safe and appropriate responses, and to inform staff about the systems currently available for trafficked people would help mental health professionals in responding to the needs of potential victims of trafficking. Further research is needed to explore the ways in which health professionals identify victims and to establish the generalizability of findings beyond this inner city mental health service.

## Additional file

Additional file 1:
**Free text terms used to search the CRIS database.** (DOCX 13 kb)

## References

[1] Abas M, Ostrovschi NV, Prince M, Gorceag VI, Trigub C, Oram S (2013). Risk factors for mental disorders in women survivors of human trafficking: a historical cohort study. BMC Psychiatry.

[2] Kiss L, Pocock NS, Naisanguansri V, Suos S, Dickson B, Thuy D (2015). Health of men, women, and children in post-trafficking services in Cambodia, Thailand, and Vietnam: an observational cross-sectional study. Lancet Global Health.

[3] Oram S, Stöckl H, Busza J, Howard LM, Zimmerman C (2012). Prevalence and risk of violence and the physical, mental, and sexual health problems associated with human trafficking: systematic review. PLoS Med.

[4] Oram S, Khondoker M, Abas M, Broadbent M, Howard LM. Characteristics of trafficked adults and childrenwith severe mental illness: a historical cohort study. Lancet Psychiatry. 2015. Online First,doi:10.1016/S2215-0366(15)00290-4 10.1016/S2215-0366(15)00290-4 26489912

[5] Zimmerman C, Borland R. Caring for trafficked persons: guidance for health providers. Geneva, Switzerland: International Organization for Migration (IOM), London School for Hygiene and Tropical Medicine (LSHTM), and United Nations Global Initiative to Fight Trafficking in Persons (UN GIFT) Retrieved from http://publications.iom.int/system/files/pdf/ct_handbook.pdf. 2009.

[6] Ross C, Dimitrova S, Howard LM, Dewey M, Zimmerman C, Oram S. Human trafficking and health: a cross-sectional survey of NHS professionals’ contact with victims of human trafficking. 2015. doi:10.1136/bmjopen-2015-008682 10.1136/bmjopen-2015-008682 PMC4550705 26293659

[7] Kliner M, Stroud L (2012). Psychological and health impact of working with victims of sex trafficking. J Occup Health.

[8] Stewart R, Soremekin M, Perera G, Broadbent M, Callard F, Denis M (2009). The South London and Maudsley NHS Foundation Trust Biomedical Research Centre (SLAM BRC) case register: development and descriptive data. BMC Psychiatry.

[9] Fernandes AC, Cloete D, Broadbent MT, Hayes RD, Chang C-K, Jackson RG (2013). Development and evaluation of a de-identification procedure for a case register sourced from mental health electronic records. BMC Med Inform Decis Mak.

[10] Braun V, Clarke V (2006). Using thematic analysis in psychology. Qual Res Psychol.

[11] Hsieh HF, Shannon SE (2005). Three approaches to qualitative content analysis. Qual Health Res.

[12] Isaac R, Solak J, Giardino AP (2011). Health care providers’ training needs related to human trafficking: maximizing the opportunity to effectively screen and intervene. J Applied Res Children.

[13] Hammen C (2005). Stress and depression. Annu Rev Clin Psychol.

[14] Miller KE, Rasmussen A (2010). War exposure, daily stressors, and mental health in conflict and post-conflict settings: bridging the divide between trauma-focused and psychosocial frameworks. Soc Sci Med.

[15] Cloitre M, Courtois C, Ford J, Green B, Alexander P, Briere J, et al. The ISTSS Expert Consensus TreatmentGuidelines for Complex PTSD in Adults. International Society for Traumatic Stress Studies. 2012. Retrieved fromhttps://www.istss.org/ISTSS_Main/media/Documents/ISTSS-Expert-Concesnsus-Guidelines-for-Complex-PTSD-Updated-060315.pdf.

[16] National Institute for Health and Care Excellence. Clinical Guideline 26. Post-traumatic stress disorder (PTSD): the management of PTSD in adult and children in primary and secondary care. London: National Institute for Health and Care Excellence., 2005. E36-49.

[17] Baldwin SB, Eisenman DP, Sayles JN, Ryan G, Chuang KS. Identification of human trafficking victims in health care settings. Health and Human Rights: An International Journal. 2011;13(1). 22772961

[18] Lederer LJ, Wetzel CA (2014). Health Consequences of Sex Trafficking and Their Implications for Identifying Victims in Healthcare Facilities. Annals Health L.

[19] Brewin CR (2007). Autobiographical memory for trauma: Update on four controversies. Memory.

[20] Dovydaitis T (2010). Human trafficking: the role of the health care provider. J Midwifery Womens Health.

[21] Grace AM, Lippert S, Collins K, Pineda N, Tolani A, Walker R (2014). Educating health care professionals on human trafficking. Pediatr Emerg Care.

[22] Riley R (2013). Identifying and referring victims of human trafficking; Training for healthcare professionals.

[23] Harrison ME, McKay MM, Bannon WM (2004). Inner-city child mental health service use: The real question is why youth and families do not use services. Community Ment Health J.

[24] Killaspy H, Banerjee S, King M, Lloyd M (2000). Prospective controlled study of psychiatric out-patient non-attendance characteristics and outcome. Br J Psychiatry.

[25] Women’s National Commission (2009). Still We Rise.

[26] Crawley H (2007). When is a child not a child? Asylum, age disputes and the process of age assessment.

