# Supplementary material for: Mental health service responses to human trafficking: a qualitative study of professionals’ experiences of providing care
Source: BMC Psychiatry. 2015 Nov 17;15:289. doi: 10.1186/s12888-015-0679-3 (PMC4650496; doi:10.1186/s12888-015-0679-3)
Supplement: Additional file 1: — Free text terms used to search the CRIS database. (DOCX 13 kb) [file 12888_2015_679_MOESM1_ESM.docx]

**Additional file 1**

Free text terms used to search the CRIS database

1. Human trafficking
2. Victim of trafficking
3. Sex trafficking
4. Child trafficking
5. People trafficking
6. Trafficking
7. Trafficked
8. Traffiked
9. Traffiked
10. Poppy Project
11. Sex traffickers
12. Forced prostitution
13. Forced labour
14. Sexual slavery
